# Supplementary material for: Fungal Infections in the ICU during the COVID-19 Pandemic in Mexico
Source: J Fungi (Basel). 2023 May 18;9(5):583. doi: 10.3390/jof9050583 (PMC10219464; doi:10.3390/jof9050583)
Supplement: Supplementary file 1 [file jof-09-00583-s001.zip › jof-2296545-supplementary.pdf]

Table S1. Characteristics of CAPA patients with clinical, radiological, and mycological data.

Case Number	General	CAPA classification	Clinical data	Radiological data	Mycological data	Outcome
**1**	Man, 24 y, SLE	Probable	Fever & worse ventilatory	Consolidations & ground glass by CT	Positive BAL GM (1.4 OD)	Live
**2**	Man, 32 y, obesity	Possible	Fever	New infiltrates in Xray	TA isolate *A. secc fumigati*	Live
**3**	Man, 33 y, obesity	Probable	Fever & worse ventilatory	New infiltrates in Xray	Positive serum GM (1.76 OD)/ TA isolate *A.* secc *fumigati*	Live
**4**	Women,38 y, obesity	Possible	Fever	New infiltrates in Xray	TA isolate *A.* secc *fumigati*	Dead
**5**	Man, 38 y, obesity	Possible	Fever & worse ventilatory	New infiltrates in Xray	TA isolate *A.* secc *fumigati*	Live
**6**	Man, 40 y, obesity	Possible	Worse ventilatory	Consolidations & ground glass by CT	TA isolate *A.* secc *nigri*	Live
**7**	Man, 44 y, obesity	Possible	Fever & worse ventilatory	New infiltrates in Xray	TA isolate A. secc *fumigati* and *A.*secc *flavi*	Dead
**8**	Man, 44 y, obesity, smoking & immunosuppression	Probable	Fever & worse ventilatory	Consolidations & ground glass by CT	BAL isolate A. secc *nigri*	Dead
**9**	Man, 45 y, overweight and HIV	Probable	Fever & worse ventilatory	Consolidations & ground glass by CT	Positive serum GM (8.7 OD)	Live
**10**	Women, 45 y, obesity and dm	Possible	Fever & worse ventilatory	Consolidations & ground glass by CT	TA isolate *A.* secc *fumigati*	Live
**11**	Man, 46 y, overweight	Probable	Fever & worse ventilatory	New infiltrates in Xray	Positive serum GM (0.8 OD)	Dead
**12**	Man, 46 y, overweight	Probable	Fever	Consolidations by CT	Positive BAL GM (4.6 OD)	Dead
**13**	Man, 47 y, overweight and hypertension	Probable	Fever	Consolidations by CT	Positive serum GM (0.723 OD)	Live
**14**	Man, 49 y, obesity, dm and hypertension	Possible	Fever & worse ventilatory	New infiltrates in Xray	TA isolate *A.* secc *fumigati*	Dead
**15**	Man, 49 y, obesity	Possible	Fever & worse ventilatory	New infiltrates in Xray	TA isolate *A.* secc *fumigati*	Dead
**16**	Man, 50 y, overweight	Possible	Fever & worse ventilatory	New infiltrates in Xray	TA isolate *A.* secc *fumigati*	Dead
**17**	Women, 52 y, overweight & dm	Possible	Fever	Consolidations by CT	TA isolate *A.versicolor*	Live
**18**	Man, 52 y, obesity, dm, hypertension, & CKD	Possible	Fever & worse ventilatory	Consolidations, micronodules & cavitation by CT	TA isolate A. secc *fumigati*	Live
**19**	Women, 53 y, obesity	Possible	Worse ventilatory	Consolidations & ground glass by CT	TA isolate *A.* secc *fumigati* and *A.*secc *nigri*	Live
**20**	Man, 53 y, overweight	Probable	Fever & worse ventilatory	Consolidations & ground glass by CT	Positive BAL GM (4.6 OD)	Live

**21**	Women, 55 y, obesity, dm	Probable	Fever & worse ventilatory	New infiltrates in Xray	Positive serum GM (2.39 OD)	Live
**22**	Man, 55 y, obesity, dm & hypertension	Possible	Fever & worse ventilatory	Consolidations & ground glass by CT	TA isolate *A.* secc *fumigati*	Dead
**23**	Man, 55 y, overweight	Possible	Fever & worse ventilatory	Micronodules by CT	TA isolate *A.* secc *fumigati*	Live
**24**	Man, 55 y, obesity	Probable	Fever	Consolidations & ground glass by CT	Positive BAL GM (6.12 OD)/ TA isolate *A.* secc fumigati	Dead
**25**	Man, 59 y, overweight & smoking	Possible	Worse ventilatory	Consolidations & ground glass by CT	TA isolate *A.* secc nidulans	Dead
**26**	Man,60 y, obesity & hypertension	Possible	Worse ventilatory	Consolidations, ground glass & micronodules by CT	TA isolate Aspergillus spp.	Live
**27**	Man,60 y, obesity & hypertension	Probable	Fever	Micronodules by CT	BAL isolate *A.* secc fumigate	Live
**28**	Women, 61 y, overweight	Possible	Fever & worse ventilatory	Consolidations & ground glass by CT	TA isolate *A.* secc fumigate	Live
**29**	Man,62 y, overweight, COPD & immunosuppression	Possible	Fever	Consolidations, ground glass & micronodules by CT	TA isolate *A.* secc terrei	Dead
**30**	Man,62 y, obesity	Possible	Fever	New infiltrates in Xray	TA isolate *A.* secc fumigati	Dead
**31**	Man,62 y, dm	Probable	Fever & worse ventilatory	Consolidations & ground glass by CT	Positive *Aspergillus* PCR	Live
**32**	Man,64 y, overweight, hypertension & immunosuppression	Possible	Worse ventilatory	Consolidations & ground glass by CT	TA isolate *A.* secc fumigati	Dead
**33**	Man,64 y, obesity	Probable	Fever & worse ventilatory	New infiltrates in Xray	Positive serum GM (0.6 OD)	Dead
**34**	Women, 66 y, overweight & hypertension	Possible	Fever & worse ventilatory	Consolidations & ground glass by CT	TA isolate *A.* secc fumigati	Dead
**35**	Women, 67 y, immunosuppression	Possible	Worse ventilatory	Consolidations by CT	TA isolate *A.* secc fumigati	Dead
**36**	Women, 67 y, obesity	Possible	Fever	Consolidations, & micronodules by CT	TA isolate *A.* secc *fumigati*	Dead
**37**	Women, 68 y, obesity, hypertension & smoking	Possible	Fever	New infiltrates in Xray	TA isolate *A.* secc nigri	Live
**38**	Women, 68 y, obesity	Possible	Worse ventilatory	New infiltrates in Xray	TA isolate *Aspergillus* spp.	Dead
**39**	Man,70 y, dm & hypertension	Possible	Fever & worse ventilatory	Consolidations & ground glass by CT	TA isolate A.secc fumigati	Dead
**40**	Women, 75 y, overweight & hypertension	Possible	Fever & worse ventilatory	Consolidations by CT	TA isolate *Aspergillus* spp.	Live

**41**	Man,75 y overweight & smoking	Possible	Fever & worse ventilatory	Consolidations & ground glass by CT	TA isolate *A.secc fumigati*	Dead
**42**	Man,77 y, overweight	Possible	Fever & worse ventilatory	Consolidations & ground glass by CT	TA isolate *A.secc fumigati*	Dead
**43**	Man,79 y, overweight, liver cirrhosis & smoking	Possible	Worse ventilatory	New infiltrates in Xray	TA isolate *A.secc fumigati*	Dead
**44**	Man, 79 y, COPD & hypertension	Probable	Worse ventilatory	Consolidations & ground glass by CT	Positive BAL GM (1.12 DO) and isolate *A.secc nigri*	Dead
**45**	Women, 81 y, obesity & hypertension	Possible	Fever & worse ventilatory	New infiltrates in Xray	TA isolate *A.secc fumigati*	Dead
**46**	Man,81 y, overweight, dm & hypertension	Possible	Fever & worse ventilatory	Consolidations & pleural effussion by CT	TA isolate *A.secc terrei*	Dead
**47**	Man, 86 y, overweight & immunosuppression	Probable	Fever & worse ventilatory	Consolidations & ground glass by CT	Positive BAL GM (10.5 OD) & TA isolate *A. secc fumigati*	Dead

BAL: bronchoalveolar lavage; CAPA: COVID-19 associated pulmonary aspergillosis; CKD: chronic kidney disease; COPD: chronic obstructive pulmonary disease; CT: computed tomography; CSF: cerebrospinal fluid; dm: diabetes mellitus; LF: lateral flow; GM: galactomannan antigen; OD: optical density index; SLE: systemic lupus erythematosus; TA: tracheal aspirate; *y*: years. *Lymphopenia was defined as total lymphocytes <1000 x 10^3/μL.
